# Successful treatment of refractory hyperferritinemic syndromes with canakinumab: a report of two cases

**DOI:** 10.1186/s12969-020-00450-9

**Published:** 2020-07-11

**Authors:** Riccardo Papa, Valentina Natoli, Roberta Caorsi, Francesca Minoia, Marco Gattorno, Angelo Ravelli

**Affiliations:** 1 Università degli Studi di Genova, Genoa, Italy; 2 IRCCS Istituto Giannina Gaslini, Genoa, Italy; 3 None, Italy; 4 Fondazione IRCCS Ca’ Granda Ospedale Maggiore Policlinico, Milan, Italy; 5 Sechenov First Moscow State Medical University, Moscow, Russian Federation

**Keywords:** Hyperferritinemic syndrome, Macrophage activation syndrome, Hemophagocytic lymphohistiocytosis, Hyperferritinemia, Anakinra, Canakinumab, Interleukin-1 inhibitors

## Abstract

**Background:**

Hyperferritinemic syndromes are systemic inflammatory disorders characterized by a dysfunctional immune response, which leads to excessive activation of the monocyte-macrophage system with hypercytokinemia and may pursue a rapidly fatal course.

**Case presentation:**

We describe two patients of 11 and 9 years of age with hyperferritinemic syndromes, one with impending macrophage activation syndrome (MAS) and one with overt MAS, who were refractory or intolerant to conventional therapies, but improved dramatically with canakinumab.

**Conclusions:**

Our report indicates that canakinumab may be efficacious in the management of hyperferritinemic syndromes, including MAS.

## Background

Hyperferritinemic syndromes (HFS) are systemic inflammatory disorders characterized by a dysfunctional immune response, leading to excessive activation of the monocyte-macrophage system with hypercytokinemia, and pronounced hemophagocytosis [1]. Serum ferritin level higher than 500 ng/ml is the common laboratory feature of this heterogeneous group of disorders, which ranges from rheumatic to non-rheumatic diseases, including primary immunodeficiencies, chronic infections, and malignancies.

The main condition within this disease spectrum is hemophagocytic lymphohistiocytosis (HLH), which is sub-classified into primary (or familial) HLH and secondary (or acquired or reactive) HLH [2]. The HLH associated with rheumatic illnesses is termed macrophage activation syndrome (MAS) [3, 4]. Among pediatric rheumatic diseases, MAS is encountered most commonly in children with systemic juvenile idiopathic arthritis (sJIA) [5]. Although many patients with active sJIA without MAS have ferritin levels exceeding 1000 ng/ml [6], when MAS develops ferritin usually increases sharply. Beside the known conditions associated with HFS, in several instances no evident cause or underlying disease is observed. Timely recognition of HFS and prompt institution of an appropriate therapy are fundamental to avoid progression toward overt MAS.

In the past two decades, several well established criteria that can help to identify MAS in its early stage have been published [7–10]. Conversely, the management of the syndrome is not standardized and no universally agreed therapeutic protocols exist. Although high-dose glucocorticoids and cyclosporine A (CSA) still represent the mainstay of the treatment, instances of MAS that are refractory to these therapies are often encountered. Recently, a number of cases of sJIA-associated MAS dramatically benefiting from the interleukin (IL)-1 receptor antagonist anakinra (ANK) after inadequate response to glucocorticoids and CSA have been reported [11–17]. However, most patients needed dose escalation, up to 10 mg/kg/day, to control symptoms [18]. Based on these data, there is now wide agreement that ANK is a valuable medication for MAS.

The role of the IL-1β antibody canakinumab (CNK) is less clear, due to both the lack of experience with its use as interventional therapy in MAS and the occurrence of instances of MAS, recorded as adverse event, in the randomized clinical trials that led to its registration in sJIA [19, 20]. However, the incidence of MAS in the trials was similar to the incidence of MAS in sJIA patients reported from a tertiary care pediatric rheumatology center in the US, which suggested that IL-1 inhibition with CNK does not have a major effect on the risk of developing MAS. In addition, many of the instances of MAS appeared to be triggered by an infection [21]. Recently, three patients with sJIA associated MAS which was refractory to conventional therapies or could not be controlled with standard doses of ANK or CNK, but responded dramatically to one single injection of CNK at higher doses (7,5 to 15 mg/kg) have been reported in a meeting abstract [22].

In the present paper, we describe two patients with HFS, one with sJIA-like illness and impending MAS and one with sJIA and overt MAS, who were resistant or intolerant to conventional therapies, but improved rapidly with the administration of CNK.

## Case presentation

### Patient 1

A previously healthy 11-year-old boy was admitted to his local hospital with a 1-week history of fever (maximum temperature 39.4 °C), urticarial rash and arthralgia, which did not improve with nonsteroidal anti-inflammatory, antihistamine and antibiotic therapy. On physical examination, he had generalized lymphadenopathy, but no evidence of overt arthritis. Body temperature was 38.7 °C. Laboratory tests showed increased acute phase reactants, anemia, and marked hyperferritinemia. Liver and kidney function tests, triglycerides, serum complement fractions, rheumatoid factor and antinuclear antibodies were all normal or negative. Abdominal ultrasound revealed diffuse lymph nodes enlargement and positron-emission tomography increased concentration of the radioactive tracer in the supraclavicular, mediastinal and abdominal lymph nodes. Chest radiograph, echocardiography, extensive infectious serology and autoantibodies were negative. Bone marrow aspirate disclosed expansion of the myeloid cell line and cervical lymph node biopsy a nonspecific reactive B and T lymphocyte hyperplasia. Next generation sequencing panel for monogenic autoinflammatory diseases and primary HLH as well as interferon signature were all negative. On the fifth day of admission, glucocorticoid therapy with 2 mg/kg/day prednisone was started, which led to defervescence of fever in 3 days and rapid improvement in clinical and laboratory abnormalities.

Six months later, after the decrease of prednisone dose to 0.15 mg/kg/day, there was recurrence of urticarial rash and diffuse arthralgia, without fever. The boy was then admitted to our hospital. Laboratory tests disclosed leukocytosis with neutrophilia, anemia, thrombocytosis, and increased erythrocyte sedimentation rate, C-reactive protein (CRP), aspartate aminotransferase (AST), lactate dehydrogenase and ferritin (Table 1, T0). Because the illness did not meet both the International League for Associations of Rheumatology (ILAR) criteria for sJIA [23], due of the absence of arthritis and fever, and the 2016 classification criteria for MAS complicating sJIA [9], due to the lack of fever, a diagnosis of systemic juvenile idiopathic arthritis (JIA)-like hyperferritinemic syndrome was made. Treatment with intravenous methylprednisolone at 3 mg/kg/day administered in three divided doses and ANK at 5 mg/kg/day (i.e. 100 mg twice a day) was initiated. However, ANK had to be first tapered to 100 mg/day and then discontinued due to the development of severe, intractable injection site reactions during the first week of treatment. In substitution of ANK, oral CSA at 4 mg/kg/day in two daily doses was started on the 7th day of hospitalization. Over the subsequent week, there was improvement in laboratory changes (Table 1, T+14) and methylprednisolone dose was, then, gradually diminished.

**Table 1:** Course of laboratory tests over time in patient 1

Days from admission at Authors’ center	0	+ 14	+ 45^a^	+ 50	+ 80
White blood cells (× 10^3^/mm^3^)	17.9	10.4	44.5	11.4	8.9
Neutrophil count (×10^3^/mm^3^)	15.0	8.9	41.7	6.4	5.0
Hemoglobin (g/dL)	10.3	10.6	9.3	9.8	12.5
Platelet count (×10^3^/mm^3^)	482	320	418	459	315
Erythrocyte sedimentation rate (mm/h)	59	37	57	61	18
C-reactive protein (mg/dL)	8.2	4.9	11.5	1.5	< 0.46
Aspartate aminotransferase (U/L)	229	68	170	127	12
Lactate dehydrogenase (U/L)	1752	951	956	862	853
Triglycerides (mg/dL)	112	158	133	226	107
Fibrinogen (mg/dL)	341	442	415	251	287
Ferritin (ng/mL)	13,200	8550	18,000	4099	581

^a^Start of canakinumab administration

However, on the 45th day of continuous hospitalization, although the boy had remained afebrile, there was worsening of his clinical conditions, with malaise, fatigue and diffuse arthralgia, and laboratory tests showed sharp increase in white blood cells, neutrophil count and ferritin, together with decreased hemoglobin and elevated AST (Table 1, T + 45). At this stage, the 2016 classification criteria for MAS were still not fulfilled, but the child was thought to have impending MAS. We, then, decided to increase methylprednisolone dose to 3 mg/kg/day and to start CNK at 4 mg/kg subcutaneously, while continuing CSA. After the first injection of CNK, there was quick and striking improvement of laboratory abnormalities, with decrease in ferritin value from 18,000 to 4099 ng/ml and normalization of white blood cell and neutrophil count within 5 days (Table 1, T + 50). One month later, the blood cell counts, the acute phase reactants and the AST had returned to normal and the level of ferritin was markedly reduced (Table 1, T + 80). The boy was, then, discharged with monthly CNK administration, in association with CSA and tapering oral prednisone.

### Patient 2

A 9-year-old girl was diagnosed with sJIA and MAS at her local hospital based on the presence of high-spiking fever, erythematous rash and polyarthritis, together with increased acute phase reactants, AST, triglycerides and ferritin, and decreased platelet count and fibrinogen. She was given high-dose intravenous methylprednisolone at 30 mg/kg for 3 consecutive days, which led to improvement in clinical manifestations and laboratory abnormalities. However, shortly after glucocorticoid therapy had been switched to oral prednisone there was a recurrence of MAS features and the girl was transferred to the regional tertiary care hospital.

On admission, a full-blown clinical and laboratory picture of MAS was detected. Three additional pulses of intravenous methylprednisolone at 30 mg/kg were, then, administered and CSA at 4 mg/kg/day, with ANK at 6 mg/kg/day, were started. This treatment led to rapid improvement in MAS features. However, during hospitalization the girl developed a thrombophlebitis in the right arm in the site of a venipuncture and developed fever, malaise and chest pain in spite of antibiotic therapy. A few days later, a chest radiograph disclosed marked cardiomegaly, which was found on echocardiography to be due to massive pericardial effusion. This complication was followed by recurrence of MAS abnormalities, with increase in ferritin to 20,350 ng/ml (Table 2, T − 4). The girl was given 3 additional pulses of intravenous methylprednisolone at 30 mg/kg and was, then, transferred to our hospital for further care.

**Table 2:** Course of laboratory tests over time in patient 2

Days from admission at Authors’ center	-4	0	+ 20	+ 30	+ 35^a^	+ 40	+ 50
White blood cells (×10^3^/mm^3^)	14.2	10.6	3.4	5.4	3.7	18.9	5.5
Neutrophil count (× 10^3^/mm^3^)	11.9	6.6	1.9	3.4	1.7	15.1	2.9
Hemoglobin (g/dL)	9.2	11.2	10.8	10.8	10.5	9.9	11.1
Platelet count (×10^3^/mm^3^)	285	424	105	233	145	164	253
Erythrocyte sedimentation rate (mm/h)	75	68	43	55	63	50	36
C-reactive protein (mg/dL)	2.5	< 0.46	1.83	< 0.46	0.6	< 0.46	< 0.46
Aspartate aminotransferase (U/L)	61	40	350	111	113	35	31
Lactate dehydrogenase (U/L)	1673	1048	4960	1541	2122	1116	468
Triglycerides (mg/dL)	209	173	273	–	157	–	–
Fibrinogen (mg/dL)	356	418	234	297	424	312	267
Ferritin (ng/mL)	20,350	5065	49,700	17,580	28,634	12,340	201

^a^Start of canakinumab administration

On admission, the girl was afebrile and laboratory tests revealed an overall improvement of MAS abnormalities as compared with previous assessment (Table 2, T0). Treatment was, then, continued with intravenous methylprednisolone at 2 mg/kg/day, together with CSA and ANK at unchanged doses. In the meantime, pericardial drainage revealed the purulent nature of the accumulated fluid, whose culture led to the isolation of a methicillin-resistant *Staphylococcus aureus*. Therapy with large-spectrum antibiotics was started. However, on the third week of hospitalization there was a sudden worsening of clinical conditions, with recurrence of fever and malaise. Repeated laboratory tests showed a full-blown picture of MAS, with sharply elevated ferritin (Table 2, T + 20), which met the 2016 classification criteria for the syndrome. Treatment consisted of 3 consecutive days of methylprednisolone at 30 mg/kg plus intravenous immunoglobulin at 2 g/kg. Cyclosporine A and ANK were continued, but the dose of the latter medication was raised to 8 mg/kg/day. The laboratory picture of MAS improved (Table 2, T + 30), but after the shift of intravenous methylprednisolone to the conventional regimen of 2 mg/kg/day and surgical pericardiotomy, which led to the evacuation of 350 ml of purulent fluid, there was another flare of MAS, with a further increase in ferritin to 28,634 ng/ml (Table 2, T + 35). Due to the presence of major signs of corticosteroid toxicity and the inefficacy of ongoing treatment in preventing MAS recurrences, we decided to start CNK at 5 mg/kg. Anakinra was stopped and shortly afterward also CSA was discontinued. The first injection of CNK was followed by prompt improvement in all MAS biomarkers and the girl could be discharged 2 weeks later in good general condition, with normal ferritin level (Table 2, T + 50) and on tapering oral prednisone. Nine months later she was receiving low-dose prednisone and monthly CNK and had experienced no relapses of either sJIA or MAS.

## Discussion and conclusions

We have described two patients with HFS, which were refractory or intolerant to conventional therapies, but responded dramatically to the administration of CNK. The first patient had a long-standing inflammatory illness closely resembling sJIA, but without overt arthritis. Although the 2016 classification criteria for MAS [9] were not met, he had a sharp increase in ferritin level, which was thought to herald the potential occurrence of full-blown MAS. Although initial ANK therapy, in conjunction with intravenous methylprednisolone and CSA, was followed by improvement in laboratory abnormalities, especially hyperferritinemia, the subsequent discontinuation of ANK because of serious and intractable injection site reaction was followed by worsening of the condition, which was reversed quickly after a single injection of CNK. The second patient had recurrent episodes of MAS, the last of which was precipitated after surgical pericardiotomy to evacuate purulent pericardial fluid and was not controlled by the combination of high-dose intravenous methylprednisolone, CSA and ANK. The administration of CNK led to rapid recovery of all clinical and laboratory features of MAS.

The rationale for the use of cytokine blockers in the management of MAS was provided by the demonstration of the prominent role of the pro-inflammatory cytokines targeted by these agents, namely TNF-α, IL-1 and IL-6, in the pathophysiology of the syndrome [4, 18]. The first cytokine inhibitors used to treat MAS were TNF blockers. However, after initial encouraging reports, subsequent experiences indicated that these agents may trigger MAS in some instances. Nowadays, TNF inhibition is not considered the ideal therapy for MAS, whereas there is much more interest for therapy directed at two other pro-inflammatory cytokines, IL-1 and IL-6 [18].

Thus far, the information on the use of IL-6 blockade in the management of MAS is still limited, given the reports from the tocilizumab clinical trials and post-marketing surveillance, which suggest that IL-6 inhibition does not provide full protection against MAS [21]. By contrast, substantial benefit from ANK treatment in sJIA-associated MAS after inadequate response to glucocorticoids and CSA has been described in many case reports [24, 25]. Notably, occurrence of MAS was occasionally seen in children treated with doses of 1–2 mg/kg daily [26, 27]. In some of these patients, however, features of MAS improved after the dose of ANK was escalated. There is now widespread consensus that ANK, particularly at higher doses, might be effective at least in some patients with sJIA-associated MAS [18].

Our report suggests that the monoclonal IL-1β antibody CNK may represent an alternative therapeutic option for children with MAS who are refractory or intolerant to conventional therapies and for select critically ill children with HFS who do not meet neither sJIA nor MAS criteria. The efficacy of CNK in ANK-resistant patients with HFS has been previously reported [28]. The better effectiveness of CNK may be due to differences in molecular targets (i.e. ANK blocks both IL-1α and IL-1β) or pharmacokinetics (i.e. longer half-life of CNK). Further clinical experience is needed to confirm our observations and to clarify whether a dose of CNK higher than the 4 mg/kg used in the routine management of sJIA is needed to control HFS, including MAS.

## Data Availability

The datasets used and/or analyzed during the current study are available from the corresponding author on reasonable request.

## Abbreviations

HFS: Hyperferrritinemic syndromes
HLH: Hemophagocytic lymphohistiocytosis
MAS: Macrophage activation syndrome
sJIA: Systemic juvenile idiopathic arthritis
ANK: Anakinra
CSA: Cyclosporine A
CNK: Canakinumab
IL: Interleukin
